# Current Development in Elderly Comprehensive Assessment and Research Methods

**DOI:** 10.1155/2016/3528248

**Published:** 2016-03-03

**Authors:** Shantong Jiang, Pingping Li

**Affiliations:** ^1^Key Laboratory of Carcinogenesis and Translational Research, Ministry of Education, Department of Integration of Chinese and Western Medicine, Peking University Cancer Hospital & Institute, Beijing 100142, China; ^2^Key Laboratory of Carcinogenesis and Translational Research, Ministry of Education, Department of Integrative Medicine and Geriatric Oncology, Peking University Cancer Hospital & Institute, Beijing 100142, China

## Abstract

Comprehensive geriatric assessment (CGA) is a core and an essential part of the comprehensive care of the aging population. CGA uses specific tools to summarize elderly status in several domains that may influence the general health and outcomes of diseases of elderly patients, including assessment of medical, physical, psychological, mental, nutritional, cognitive, social, economic, and environmental status. Here, in this paper, we review different assessment tools used in elderly patients with chronic diseases. The development of comprehensive assessment tools and single assessment tools specially used in a dimension of CGA was discussed. CGA provides substantial insight into the comprehensive management of elderly patients. Developing concise and effective assessment instruments is helpful to carry out CGA widely to create a higher clinical value.

## 1. Introduction 

Since the 21st century, the aging of the population began to accelerate. Although the aging of the population is still concentrated in developed countries, many developing countries have entered the era of an aging population. The United Nations predict that population aging will occur mainly in developing countries in the next 35 years [1]. The World Health Organization (WHO) categorized 60–74-year-olds as the younger elderly, 75–89-year-olds as the elderly, and ≥90-year-olds as elderly elderly or macrobian elderly, while the National Comprehensive Cancer Network (NCCN) defined the 65–75-year-olds as the younger elderly, 76–85-year-olds as the elderly, and >85-year-olds as the macrobian elderly [2].

According to the assessment in 2010, the balance of the global burden of disease is gradually tilted to chronic diseases which will be the first burden of the global elderly. The most common chronic illnesses include cardiovascular disease, heart disease, cancer, chronic respiratory diseases, musculoskeletal disorders, pulmonary disease, diabetes, cognitive impairment, and depression, among which cardiovascular disease is a major killer of elderly health, especially ischemic heart disease. As Beard and Bloom [3, 4] make clear in their viewpoint, increased disease burden will be mainly concentrated in those age-related diseases. For example, due to the aging of population, Alzheimer's disease will further increase the burden on the elderly in the next 30 or 40 years. The latest estimates show that the number of patients with dementia is expected to increase from the current 44 million to 135 million by 2050. Therefore, under the new situation of the elderly increasing demand for health services, it is an inevitable requirement and a challenge to develop some new models and innovative elderly disease control to achieve healthy aging.

Comprehensive Geriatric Assessment (CGA) was proposed by Warren in the late 1930s. The National Institutes of Health (NIH) organized experts in relevant disciplines to develop the standard of CGA in 1987 [5, 6]. CGA provides detailed information on clinical, functional, and cognitive domains of older patients; it concerns the general health of the elderly and multidimensional and comprehensive scientific assessment of health status. CGA is an important way to implement the comprehensive management of aging populations. It integrates physical health, mental health, functional status, social adaptability, and environment conditions and quantifies the elderly overall health objectively. Not only is CGA an assessment, but also it formulates and makes treatment plans that protect the health and functional status of the elderly to maximize their quality of life (QoL). CGA has become an important means of the management of the elderly [7–9].

## 2. The Contents of CGA and the Assessment Tools Used in Each Domain

### 2.1. The Overall Functional Status, including Physical Health, Activities of Daily Living, and Fall Risk Assessment

The World Organization of National Colleges, Academies and Academic Association of General Practitioners/Family Physician (WONCA) considers that separate evaluation could not reflect the actual function of a whole person or his/her activities of daily living, though modern medicine has its own criteria for evaluating the function of each organ system.

The basis of CGA is comprehensive functional assessment, which should include lots of elements, such as the situation of disease of the elderly, hearing, vision, and suffering from urinary incontinence. Frailty is a state of vulnerability to poor resolution of homoeostasis after a stressor event and is a consequence of cumulative decline in many physiological systems during a lifetime. Frailty is an important geriatric syndrome linked to increased mortality, morbidity, and falls risk. A longitudinal assessment for a period of two years by Ng et al. [10] assessed 1685 Singaporean elderly with Frailty Risk Index (FRI). Weakness, slowness, low physical activity, weight loss, and exhaustion are included in FRI, and evaluation is rated on seven levels (very healthy, healthy, in good health, surface weakness, mild weakness, moderate weakness, and severe weakness) [11]. The results of their study demonstrated that FRI with a certain degree of reliability and validity as a tool is applied in predicting frailty symptoms of the elderly and decline in functional status. A systematic review on the reliability and validity of FRI also proved it [12].

Currently, functional status was measured by activities of daily living (ADL) and instrumental activities of daily living (IADL) [13]. Barthel's Index Rating Scale is the most commonly used for ADL with the total score of 100 points, with assessment based on scoring criteria. The Katz Index of Independence in Activities of Daily Living, commonly referred to as the Katz ADL, summarizes overall performance in bathing, dressing, going to toilet, transferring, continence, and feeding. Clients scored yes/no for independence in each of the six functions; grading is based on A~G seven functional levels, where higher level indicates lower ADL [14–16]. Function Activity Questionnaire (FAQ) is the preferred rating scale of IADL; the higher the score, the more severe the disorders, with score of more than 5 considered as abnormal. Rapid Disability Rating Scale (RDRs) is also an assessment tool for IADL, which is used for hospitalized and community-dwelling patients, particularly appropriate for elderly patients, but rarely used in clinical practice. The degree of help needs of daily life, degree of disability, and the degree of special issues are taken into account with the highest score of 54, where higher score indicates more severe disability.

In addition, falling fracture always occurs in the patients with balance and gait disorder, with fall rates as high as 50% under the age of more than 80 years, of which more than half of the elderly falls had occurred several times [17]. Fall was the third cause of chronic disability in the elderly which can lead to fractures, soft tissue damage, brain damage, and death [18].

There are many methods and scales for balance and fall risk assessment, summarized in Table 1. Berg Balance Scale (BBS) is the world's balance scale for patients with stroke and showed great reliability, validity, and sensitivity in different recovery stages of stroke [19, 20]. BBS assessed balance and fall risk with standing, turning around, standing on one leg, and a total of 14 other actions, with scores ranging from 0 to 56 with the cutoff point as 45, where lower score indicates higher fall risk. According to Pereira's study [21], BBS was better than the posturographic Balance Stability System (BSS) in elderly fall risk assessment. Timed Up and Go Test (TUGT) [22] and Tinetti Gait and Balance Test [23] are widely used to measure the functional activity of the elderly balance and physical fitness, while the latter can also be used to predict the fall risk by testing the patient's gait and balance function. The Fall Risk Assessment Scale for the Elderly (FRASE) is correlated with St. Thomas's Risk Assessment Tool (STRATIFY) [24]; both of them have the disadvantage of containing only internal fall factors, but STRATIFY is more detailed. The Fall Risk Index (FRI) [25] is suitable for patients with stroke, and elderly patients with physical and cognitive impairment falls risk assessment usually use the Fall Assessment Tool (FAT), which includes assessment of fall-related environment factors. Therefore, FAT is also suitable for elderly patients newly admitted to assess the fall risk factors due to environmental changes.

Erik Stone reported a low-cost, continuous, environmentally mounted monitoring system, average in-home gait speed (AIGS) [26], compared to a set of traditional physical performance instruments. The results indicate that AIGS is able to predict how an individual would score on all traditional instruments and that the observed and smoothed values of AIGS show better agreement than those of any of the traditional instruments [27]. However, Gilles [28] assessed fall risk of 380 elderly; the results of that study indicated that multiple modes of gait evaluation provide a more comprehensive mobility assessment than one assessment alone and better identify incident falls in the elderly.

### 2.2. Cognitive Function

Dementia is a common cause of disability in the elderly; 50%–70% of dementia cases are Alzheimer's disease (AD). Mild cognitive impairment (MCI) is a known precursor to Alzheimer's disease. However, MCI is often overlooked and attributed to aging rather than being investigated [29]. Not only will it affect the tolerance to treatment, but also it would undermine gains of treatment in the presence of cognitive impairment in elderly patients.

Mini-Mental State Examination (MMSE) [38] and Montreal Cognitive Assessment (MoCA) are two common tools used in cognitive function assessment. MMSE is administered in 5–10 minutes and functions including registration, attention, calculation, recall, language, ability to follow simple commands, and orientation are examined. Lower score indicates more severe impairment. But MMSE has the disadvantage of being influenced by patient's education, economic status, and other factors and difficulty recognizing MCI [29]. MoCA assesses several cognitive domains, including visuospatial abilities, multiple aspects of executive functions, attention, concentration, working memory, and language. The test is available in 35 versions, each of which has its own evaluation standard [39–43]. The test and administration instructions are freely accessible for clinicians at http://www.mocatest.org/. Studies [60, 61] have shown that, compared to MMSE, MoCA covered more cognitive domains and had a higher efficiency to assess MCI. In addition, there are the Informant Questionnaire on Cognitive Decline in the Elderly (IQCODE) [44] and simple clock drawing test (CDT).

### 2.3. Emotional and Psychological Conditions

Series of complex emotional psychological problems that greatly affect the occurrence, development, and treatment of diseases will occur to the elderly as a result of dysfunction or sudden changes of living environment. And of them, depression has been known to be associated with functional limitations in elderly populations, while anxiety is often overlooked by the focus on dementia and depression, receiving little attention even though it has occurred in the elderly [62]. It is extremely necessary to identify and confirm depression and anxiety as early as possible.

The Geriatric Depression Scale (GDS) is a 30-item self-report assessment specially used to identify depression in the elderly developed in 1982 by Yesavage et al. [45, 46]. A simple version of GDS, GDS-5 (short version 5-item Geriatric Depression Scale), has been referred [47]. The GDS questions are answered “yes” or “no” for depression, reduced activity, irritability, withdrawal, painful thoughts, and negative evaluation of the past, present, and future. The grid sets a range of 20–30 as “severely depressed,” 10–19 as “mildly depressed,” and 0–9 as “normal.” The Center for Epidemiologic Studies Depression Scale (CES-D) [48] is a short self-report questionnaire with 20 items that reflect depression severity in depressed mood, feelings of guilt and worthlessness, feelings of helplessness and hopelessness, psychomotor retardation, loss of appetite, and sleep disorders, scoring the frequency of occurrence of specific symptoms during the previous week on a four-point scale at total scores of 60 and scoring ≥6 as CES-D depression. Higher scores indicate more seriousness. CES-D is not suitable for assessing the changes in the severity of depression in the course of treatment.

The Hamilton Rating Scale for Depression (HRSD) [49], also named HDRS or HAMD, is a multiple item questionnaire used to provide an indication of depression designed by Hamilton in 1960, which is the most classic and widely used scale to rate the severity and changes of adults' depression by probing mood, feelings of guilt, suicide ideation, insomnia, agitation or retardation, anxiety, weight loss, and somatic symptoms. A score of 0–7 is considered to be normal. Scores of 20 or higher indicate moderate, severe, or very severe depression and are usually required for entry into a clinical trial. Currently, another four versions were developed to include up to 29 items (7, 21, 24, and 29 items) except for the original 17-item version [63].

The GAI (Geriatric Anxiety Inventory) [50] and the GAS (Geriatric Anxiety Scale) [51] are specially developed for the elderly to assess anxiety symptoms over the past week. Each of the GAI's 20 items is rated “agree” or “disagree.” Higher scores indicate greater anxiety symptoms. However, as Gould et al. [64] described, the factor which strongly associated with anxiety is depressed status of elderly patients rather than age when assessed by GAI and GAS, which suggests that GAI and GAS are also suitable for the assessment of the nonelderly. The 30-item GAS measures anxiety severity in somatic, cognitive, and anxiety symptoms. The team of GAI developed a short form of the Geriatric Anxiety Inventory (GAI-SF) in 2011 [52], which was confirmed to have the same validity and reliability as GAI [65]. In addition, the Diagnostic and Statistical Manual of Mental Disorders (DSM), published by the American Psychiatric Association (APA), can also be used to assess anxiety. The newest DAM-5 was published in 2013 [53].

### 2.4. Nutrition Status

Malnutrition, a major problem associated with the elderly, especially elderly hospitalized patients, affects the immune and organ function and has an extensive impact on mortality and morbidity [66, 67].

Many nutrition screening tools are available for malnutrition identification. The Subjective Global Assessment (SGA) [54] is a tool to assess nutrition status developed by Detsky et al. in 1987, recommended by ASPEN (American Society for Parenteral and Enteral Nutrition), performed based on patients' medical history and physical examination. It asked participations to record changes in weight, dietary intake, functional capacity, gastrointestinal symptoms, metabolic stress, loss of subcutaneous fat, muscle wasting, and ankle/sacral edema, instead of anthropometric and biochemical tests. Assessment according to the number of the levels (A, B, and C) above 8 projects (5 of which belong to B or C, resp.) indicates moderate or severe malnutrition. It has advantage of simple operation, repetitiveness, and no need for any biological molecule, whereas it may be not accurate because the assessment is based on the subjective impression [68].

The Mini-Nutritional Assessment (MNA) [55, 56] is an elder-special tool and is extensively validated in nutritional risk screening and nutritional status assessment. It includes 18 questions in four domains: nutritional assessment, subjective assessment, anthropometric assessment, and general assessment. With a total score of 30, scoring ≥24 indicates good nourishment, scoring 17–24 indicates risk of malnutrition, and scoring <17 indicates malnutrition. Dent et al. [69] evaluated the ability of MNA for elderly inpatients to predict clinical six-month outcome, and the results show that it was still a useful predictor of poor six-month outcome, although with low accuracy. A simpler version of the MNA, the short-form Mini-Nutritional Assessment (MNA-SF) developed by Rubenstein in 2001, to be further revised by Kaiser et al. in 2004 [57], has great correlation with MNA and is widely used to screen nutritional status of the population. Currently, two versions of MNA-SF are available: MNA-SF-BMI (body mass index) and MNA-SF-CC (calf circumference).

The Geriatric Nutritional Risk Index (GNRI) has been developed as a screening tool after Nutritional Risk Index (NRI) on the basis of improving the defect that it is difficult to determine the past weight of the elderly to assess the nutritional risk. It calculates weight according to the Lorentz formula (WLo): men = *H* − 100 − [(*H* − 150)/4]; women = *H* − 100 − [(*H* − 150)/2.5]; men: *H* (cm) = [2.02 × KH  (cm)]−[0.04 × age  (y)] + 64.19; women: *H* (cm) = [1.83 × KH  (cm)]−[0.24 × age  (y)] × 84.88 (*H*: height, KH: knee height). And then we can obtain 3 segments of GNRI according to the formula GNRI = (1.489 × albumin (g/L)) + (41.7 × (weight/WLo)): severe risk (scores < 82); moderate risk (82 ≤ GNRI ≤ 92); low risk (92 ≤ GNRI ≤ 98); and no risk (scores > 98). Research has shown that GNRI can also be a useful predictor of poor six-month outcome besides mortality [69].

## 3. The Research of Comprehensive Geriatric Assessment Tools

Comprehensive assessment tools can be used directly in specific implementation in CGA [70]. Currently, a large number of comprehensive assessment tools have been established for the elderly (including healthy elderly and elderly patients), several of which are suitable for a comprehensive assessment of the general level of health (e.g., OARS, older American resources and services; CARE, Comprehensive Assessment and Referral Evaluation) in elderly patients or for the quality of life (QoL) in elderly patients (e.g., Geriatric QoL Questionnaire (GQLQ), Quality of Life Cards (QLC)); common comprehensive assessment tools were summarized in Tables 1 and 2.

The OARS is the first comprehensive tool to assess general health of the elderly developed by Duke University Center for the study of aging and human development in 1975. The OARS is the most widely used tool, which has been used for the longest time, and covers a pool of domains, whose reliability and validity have been extensively validated [70, 71]. The OARS multidimensional functional assessment questionnaire (OMFAQ) was included in the OARS, which is used to carry out assessment in 5 domains: physical health, activities of daily living, mental health, social resources, and economic resources of the elderly. Scoring of each item as 6 points and assessing the comprehensive health status of the elderly according to the score [72] are carried out. About the instructions of the OARS, the Duke Center even offers specialized training. The Comprehensive Assessment and Referral Evaluation (CARD) is a 1500-item assessment tool established by Gurland and Kuriansky in 1977 [70, 73]. It summarized medical, mental, nutritional, economic, and social health to record, classify, and grade the general health and social health of the elderly.

The Philadelphia Geriatric Centre Multilevel Assessment Instrument (PGCMAI) was primarily developed in 1982 [13]. And with the founder modification continually, the mobility was integrated into the questionnaire. Until 1983, the final complete version of the PGCMAI systematically assesses behavioral competence in the domains of physical health, cognition, activities of daily living, time use, mobility, social interaction, and environmental conditions. This model divides the content of each field by rank from simple to complex. Physical health, for example, will be assessed in accordance with the cell, tissue, and organ on all levels. Depending on the number of questions included, there are 3 versions of PGCMAI (101Q, 67Q, and 48Q, where Q means questions).

The LEIPAD is a questionnaire to assess QoL in the elderly which was developed by De Leo et al. in conjunction with the European office of the World Health Organization [30]. The latest version of the questionnaire is composed of 49 self-assessment items including the domains of physical function, personal construction, depression and anxiety, cognitive functioning, sexual functioning, and life satisfaction. De Leo et al. established reliability and validity of the tool in several languages and evaluated it for cultural competency during the development and testing of the instrument.

The WHOQOL-BREF (World Health Organization Quality of Life-BREF) is a 26-item short model from the 100-item WHOQOL for assessing the QoL in the elderly. Domains included in this tool are physical and mental health and social and environmental domain. The WHOQOL-BREF is a generic tool used either in the elderly or in a population of a specific disease as a QoL screening assessment. It is being used in populations of chronic liver and pulmonary disease to collect data on health and QoL throughout the United States and in other industrial nations [31].

An English version of concise screening tool named “Dr. SUPERMAN” for CGA was reported by Iwamoto et al. in 2013 [32]. Included in the questionnaire are physical domain, nutritional domain, psychological domain, and activities of daily living. Participants were asked to select the appropriate records related to their situations. As described in the article, “Dr. SUPERMAN” is simple to operate, but the completion time depends on the communication and understanding of patients, overall about 5–15 minutes. “Dr. SUPERMAN” was originally designed by scholars from Japan. Iwamoto et al. established reliability and validity of the scale in a population of chronic diseases, including Alzheimer's disease, osteoarthritis, cerebrovascular disease, depression or anxiety, and cardiopulmonary disease. No research has been reported on reliability and validity of the English version.

The contents of comprehensive assessment tools used in CGA are all too complicated, and even the most comprehensive OARS scale also failed to cover all the domains and information about the health function of the elderly. Besides, CGA with time-consuming consultation led to difficulty in clinical practice. As a result, some single assessment tools mentioned above combine to comprehensive use in CGA, and that was the means in a study by Avelino-Silva et al. [33] to predict the mortality and adverse outcomes in elderly patients and obtain reliable results, which also fully demonstrated the significance of the application of CGA.

## 4. Conclusion 

Comprehensive geriatric assessment is a core and an essential part of the comprehensive management of the elderly. With its increasing value, applications of CGA are increasingly widespread, including prediction of adverse outcomes of chronic diseases [33], applications in elderly cancer patients [74, 75], and being recommended in the preoperative evaluation [76]. All the evidence suggests that they will benefit from CGA both in the healthy individuals and in those with significant impairments and multiple comorbidities. CGA can be carried out throughout the entire process of elderly patients with chronic diseases, which especially helps identify some potential comorbidities that may affect clinical decision-making, treatment outcomes, and QoL in elderly patients.

The development of CGA is of great significance, but it is more important to evaluate the validity and reliability of the assessment tool, which is the premise of the effective implementation of CGA. As described in this text, there are a lot of tools used in CGA, including the comprehensive assessment scales and single assessment tool focusing on a special domain of aging populations with different characteristics. Even though the comprehensive use of the subscale has a good effect, there are still some differences between the implementers in choices of scales. In addition to the effectiveness, reliability, and other basic elements, a good scale should be more concise but it should involve enough comprehensive core assessment domains, be easier to operate and understand, be less time-consuming, and be more economic with advances in technology. Meanwhile, a special-developed scale for every single elderly common chronic disease, such as chronic ischemic heart disease and dementia, will contribute to more efficient management of elderly patients, and this is the challenge and the direction of our future efforts.

## Figures and Tables

**Table 1:** Screening instruments used in comprehensive geriatric assessment about elderly patients.

Dimension	Contents	Common single measurement tool	Common comprehensive tool
Overall functional status			
Physical health	Reserves and endurance	FRI (Frailty Risk Index) [11];	OARS (older American resources and services) [30] CARE (Comprehensive Assessment and Referral Evaluation) [31] PGCMAI (Philadelphia Geriatric Centre Multilevel Assessment Instrument) [32] LEIPAD [33] WHOQOLBREF (the World Health Organization Quality of Life-BREF)
		Fried frailty instrument [34];	
		Gill frailty instrument [34]	
		
	Situation of disease	General questionnaire	
		
	Comorbidities and the severity	CIRS-G (Cumulative Illness Rating Scale for Geriatrics);	
		CCI (Commodity Channel Index)	
		
Activities of daily living	Physical living activity status	General questionnaire	
		
	Physiological activities of daily living (ADL)	ADL Index:	
		Barthel's Index Rating Scale, Katz ADL [14–16];	
		PULSES; revised Kenny Score	
		
	Instrumental activities of daily living (IADL)	IADL Index:	
		FAQ (Function Activity Questionnaire);	
		RDRS (Rapid Disability Rating Scale)	
		
	Balance and fall risk	BBS (Berg Balance Scale) [19, 20];	
		TUGT (Timed Up and Go Test) [22];	
		Tinetti Gait and Balance Test [23];	
		FRASE (Fall Risk Assessment Scale for the Elderly) [24];	
		STRATIFY (St. Thomas's Risk Assessment Tool) [24];	
		FRI (Fall Risk Index) [25]; FAT (Fall Assessment Tool) [35];	
		MFS (Morse Fall Scale) [36]; Hendrich II Fall Risk Model [37]	
		Other tools: One-Leg Balance Test, Physical Performance Test, and Multidirectional Reach Test	
Mental and psychological condition	Cognitive function	MMSE (Mini-Mental State Examination) [38];	
		MoCA (Montreal Cognitive Assessment) [39–43];	
		IQCODE (Informant Questionnaire on Cognitive Decline in the Elderly) [44]; CDT (simple clock drawing test)	
		
	Depression	GDS (Geriatric Depression Scale) [45, 46];	
		GDS-5 (short version 5-item GDS) [47];	
		CES-D (Center for Epidemiologic Studies Depression Scale) [48];	
		HRSD (Hamilton Rating Scale for Depression) [49]	
		
	Anxiety	GAI (Geriatric Anxiety Inventory) [50];	
		GAS (Geriatric Anxiety Scale) [51];	
		GAI-SF (short form of GAI) [52];	
		DSM-5 (Diagnostic and Statistical Manual of Mental Disorders) [53]	
		
Nutritional status	Evaluation of malnutrition	SGA (Subjective Global Assessment) [54];	
		MNA (Mini-Nutritional Assessment) [55, 56];	
		MNA-SF (short-form Mini-Nutritional Assessment) [57];	
		GNRI (Geriatric Nutritional Risk Index) [58];	
		NRS-2002 (Nutrition Risk Screening 2002) [59]	
		
Social health	Demand of social support	General questionnaire	
		
Economic and environmental condition conditions	Economic conditions and medical resources;	General questionnaire	
	home security		
	transport and communications		

**Table 2:** Instrument domains.

Domain	OARS	CARE	PGCMAI	LEIPAD	WHOQOLBREE
Physical function	×	×	×	×	×
Activities of daily living	×		×		
Cognitive functioning	×		×	×	×
Psychological well-being	×	×	×	×	×
Nutritional status		×			
Social well-being	×	×	×	×	×
Financial	×	×			
Environmental			×	×	×
Sexual function				×	
Personal construct^*∗*^				×	
Life satisfaction				×	

^*∗*^Personal construct psychology (PCP) is a theory of personality and cognition developed by Kelly in the 1950s which stated that each individual's task in understanding their personal psychology is to put in order the facts of his or her own experience.
